# ANCA-associated vasculitides in childhood

**DOI:** 10.1186/1546-0096-9-S1-P95

**Published:** 2011-09-14

**Authors:** Diána Garan, Zsófia Bence, Zsófia Gács, András Szabó, Tamás Constantin

**Affiliations:** 1 2nd Department of Paediatrics, Semmelweis University, Hungary

## Background

Systemic vasculitides, especially anti-neutrophil cytoplasmic antibody (ANCA) -associated diseases are comparatively infrequent in childhood. In order to prevent significant morbidity and mortality identification is of high importance in time. New classification criteria of childhood vasculitides can facilitate making an early diagnosis.

## Aim

Here we present two female patients suffering from two different types of ANCA-associated vasculitis.

## Methods and result

14-year-old patient diagnosed with TGA at birth, at the age of 14 she presented a sudden hepatosplenomegaly. The systematic examination confirmed WG. According to our knowledge this is the first case of association of these two infrequent diseases. Furthermore hepatosplenomegaly as initial symptom of WG is especially rare feature.

The second patient suffering from asthma for years became non-responder for adequate bronchodilator therapy. Laboratory tests revealed eosinophilic leukocytosis. After excluding the most frequent causes of eosinophilia, the biopsy of maculopapular and nodular skin lesions showed vasculitis, and ANCA antibodies were found, we diagnosed Churg-Strauss syndrome.

## Conclusion

The first case highlights that ANCA associated vasculitis can be presented with uncommon symptoms. Churg-Strauss syndrome is extremely rare in childhood, outcome of the disease is unknown, and the authors urge further investigations in order to describe pediatric characteristics. Although the spectrum of symptoms is broad, being a rarity childhood vasculitis still is a challenge for physicians.

